# A Promising Proton Conducting Electrolyte BaZr_1-*x*_Ho_*x*_O_3-δ_ (0.05 ≤ *x* ≤ 0.20) Ceramics for Intermediate Temperature Solid Oxide Fuel Cells

**DOI:** 10.1038/s41598-020-60174-4

**Published:** 2020-02-26

**Authors:** Deepash S. Saini, Avijit Ghosh, Shuvendu Tripathy, Aparabal Kumar, Sanjeev K. Sharma, Nawnit Kumar, Shubhankar Majumdar, Debasis Bhattacharya

**Affiliations:** 1 Department of Physics, Deen Dayal Upadhaya Gorakhpur University, Gorakhpur, 273009 India; 2 Department of Physics, Central University of Jharkhand, Ranchi, 835205 India; 3 Materials Science Centre, Indian Institute of Technology, Kharagpur, 721302 India; 4 Department of Physics, Malti Dhari College, Naubatpur, Patliputra University, Patna, 801109 India; 5 Department of Electronics and Communication Engineering, National Institute of Technology, Meghalaya, 793003 India

**Keywords:** Fuel cells, Fuel cells

## Abstract

In this study, the Ho-substituted BaZrO_3_ electrolyte ceramics (BaZr_1-*x*_Ho_*x*_O_3-δ_, 0.05 ≤ *x* ≤ 0.20) were synthesized through a low-cost flash pyrolysis process followed by conventional sintering. The effects of Ho-substitution in BaZrO_3_ studied in terms of the structural phase relationship, microstructure and electrical conductivity to substantiate augmented total electrical conductivity for intermediate temperature solid oxide fuel cells (IT-SOFCs). The Rietveld refined X-ray diffraction (XRD) patterns revealed that pure phase with $Pm\bar{3}m$ space group symmetry of cubic crystal system as originated in all samples sintered at 1600 °*C* for 8 *h*. The Raman spectroscopic investigations also approved that Ho incorporation in BaZrO_3_ ceramics. Field Emission Scanning Microscopic (FESEM) study informed a mixture of fine and coarse grains in the fracture surface of Ho-substituted BaZrO_3_ sintered samples. The relative density and average grain size of samples were observed to decrease as per the addition of Ho-substitution in BaZrO_3_ ceramics. The electrical conductivity study was accomplished by Electrical Impedance Spectroscopy (EIS) under 3% humidified O_2_ atmosphere from 300 to 800 °*C*. Furthermore, the total electrical conductivity of BaZr_0.8_Ho_0.2_O_3-δ_ ceramic was found to be 5.8 × 10^−3^ *S-cm*^*−*1^ at 600 °*C* under 3% humidified atmosphere, which may be a promising electrolyte for IT-SOFCs.

## Introduction

Recently, the proton conductive oxide ceramics have fascinated worldwide attention due to widespread applications in intermediate temperature solid oxide fuel cells (IT-SOFCs), hydrogen separation and electrolysis of steam, etc. In this context, the rare-earth cerates and zirconates with the perovskite-type A(II)B(IV)O_3_ crystallographic structure are the two foremost families of proton-conducting oxides for electrochemical applications^1–4^. Generally, in these categories of oxide materials, oxygen vacancies are increased by replacement of tetravalent cation B(IV) by trivalent cation M(III) as given in the Eq. (1) using Kröger-Vink  notation.

1 $$2{B}_{B}^{x}+{O}_{O}^{x}+{M}_{2}{O}_{3}\to 2{M{\prime} }_{B}+{V}_{O}^{\bullet \bullet }+2B{O}_{2}$$

In this case, an H_2_O molecule from the vapor phase dissociates into hydroxide ($O{H}^{-}$) ion and proton (${H}^{+}$) in these oxide materials. The hydroxide ($O{H}^{-}$) ion fills up an oxygen vacancy, and proton (${H}^{+}$) forms a hydroxide ($O{H}^{-}$) ion with oxygen lattice^5–7^ according to the Eq. (2).

2 $${H}_{2}O(g)+{V}_{O}^{\bullet \bullet }+{O}_{O}^{x}\to 2O{H}_{O}^{-}$$

The protons are induced in these types of the perovskite oxides through oxygen vacancies due to replacement of tetravalent cation B(IV) by trivalent cation M(III). This proton conducts through hopping to the adjacent oxygen site and revolving around the oxygen that contribute to the protonic conductivity in the material^6,7^. Furthermore, trivalent cation M(III) substituted barium zirconates (i.e. BaZr_1-*x*_M_*x*_O_3-δ_) are more chemically stable than the typical proton-conducting barium cerate and have decent proton conductive activity under a wet atmosphere^8,9^.

Furthermore, the trivalent cation substituted BaZrO_3_ powders are generally synthesized by the solid-state reaction method through oxide or carbonate precursors. In addition to that, several wet chemical methods have also been employed to prepare the above powders, such as co-precipitation^10^, modified Pechini^11^, glycine-nitrate^12,13^, sol-gel^14,15^, polyacrylamide gel^16^, molten salt^17,18^, and hydrothermal methods^19,20^, etc. All these ways, contemporary afford their merits and demerits. In the sol-gel process, the rate of hydrolysis of different types of metal alkoxide limits its usage, while, inhomogeneity in compositional distribution and agglomeration of particles are the main bottlenecks in co-precipitation method^21^. Furthermore, inhomogeneous particle size, as well as irregular morphology in hydrothermal route, restricts the use of this process. An alternative way, a high temperature ( > 1400 °*C*) is required to synthesize single-phase material considerable a large size ( > 100 *nm*) of particle. However, the oxide nano-particles are desirable for good sinterability with large grain size and reflect high conductivity of perovskite material^22^. Therefore, from the perspective of synthesis, it is still an open challenge to prepare the oxide nano-particles at a relatively low temperature.

Other than the above, the substitution of a trivalent cation is recurrently exploited to escalation the conductivity of zirconates. The numerous factors, such as electronegativity, ionic radius, and so on, should be considered in a sophisticated way of choosing the substituent^23,24^. Trivalent elements include rare-earth elements, transition elements, and several main groups of elements, such as Y^3+^, Gd^3+^, Yb^3+^, Dy^3+^, Sm^3+^, In^3+^, Sc^3+^, etc^25,26^. are typically used as a substituent for zirconates. Recent theoretical investigations have revealed that the ionic radius of substituent directly controls substituent-proton interaction energy. Furthermore, the ionic radius of substituent directly controls the trapping effect of protons by a substituent, also. The trapping effect of protons by substituent is minimum for Y (90 *pm*) substituted BaZrO_3_ ceramics^23,24^. In this context, Y-substituted BaZrO_3_ ceramics have engrossed prodigious attention because of its effective proton conductivity and many researchers are also working on it^9,27,28^. But, some researcher reported very little proton conductivity for trivalent substituted BaZrO_3_^9,27–30^. However, Kreuer *et al*.^30^ demonstrated that single crystal of Y-substituted BaZrO_3_ had reasonably high proton conductivity (5 × 10^−5^ *S-cm*^*−1*^) even at 140 °*C*. Then Bohn and Schober^9^ established the enormous proton bulk conductivity (3 × 10^−3^ *S-cm*^*−1*^) of it in a wet hydrogen atmosphere at 600 °*C*. Furthermore, Han *et al*. reported a remarkably large conductivity of Dy-substituted BaZrO_3_ under humidified reducing environment, which is reduced unusually under oxidizing atmosphere because of instability in oxidization state of Dy from 3+ to 4+ state^31^. Similarly, Ahmed *et al*. presented total conductivity of 1 × 10^−4^ *S-cm*^*−1*^ for Yb-substituted BaZrO_3_ at 600 °*C* under wet air^32^ and also conductivity of 1 × 10^−4^ *S-cm*^*−1*^ for hydrated In-substituted BaZrO_3_ at 600 °*C*^33^. But, according to theoretical studies, the Ho-substituted BaZrO_3_ may support to progress the conductivity and explore the new substituent for BaZrO_3_ regarding proton-conducting electrolyte since the ionic radius of Ho^3+^ (90.1 *pm*) is very similar to that of Y^3+^ (90 *pm*)^34^. However, the study about Ho-substituted BaZrO_3_ has been little consideration in the scientific world so far^34–36^. In this work, we adopted Ho as a single substituent for BaZrO_3_ ceramics to explore the influence of Ho^3+^ on the electrical properties of BaZrO_3_ ceramics for operative SOFC applications.

## Results and Discussion

### Structural and microstructural behaviours of BaZr_1-*x*_Ho_*x*_O_3-δ_ (0.05 ≤ *x* ≤ 0.20)

**Figure 1:**
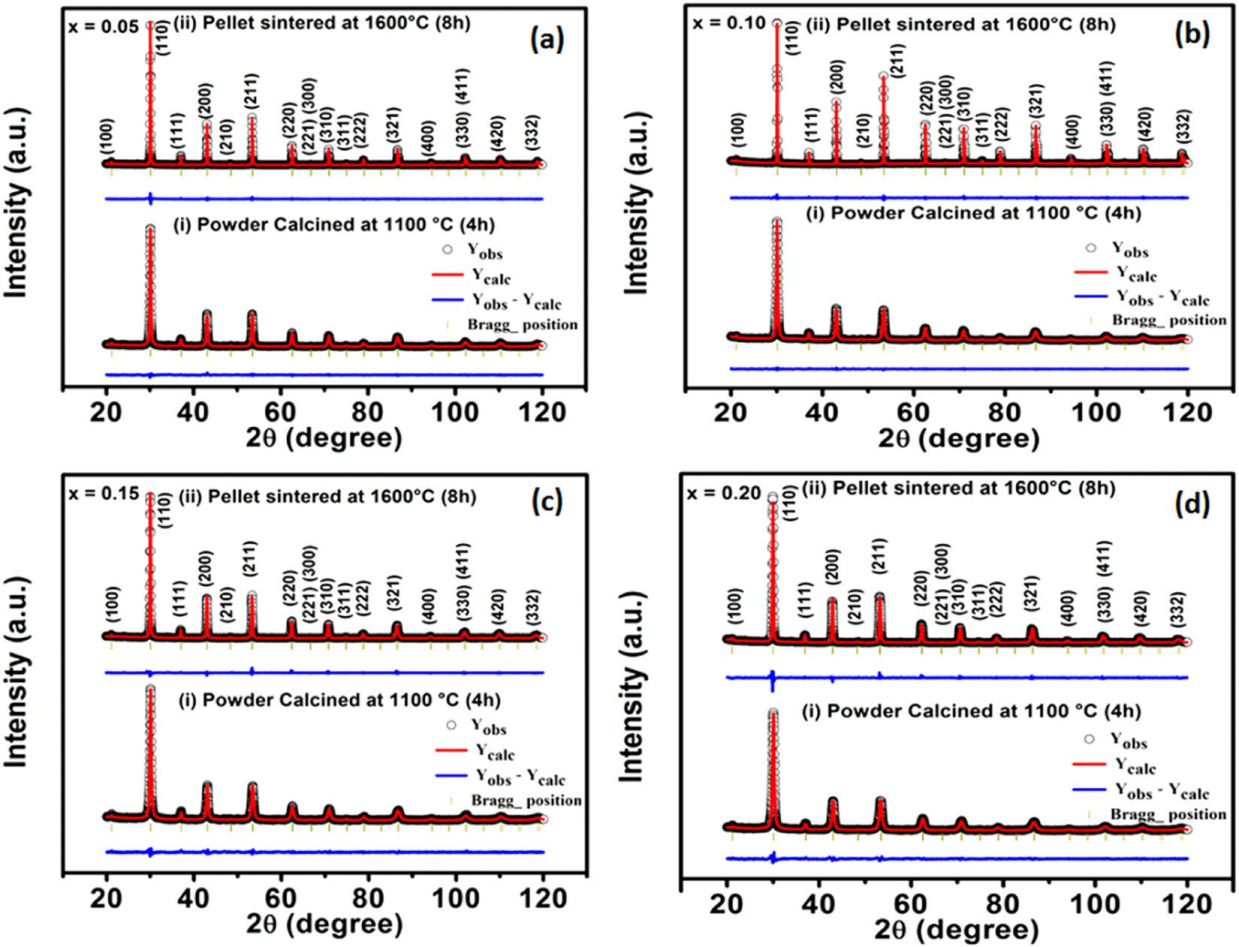
Rietveld refined XRD patterns of BaZr_1-*x*_Ho_*x*_O_3-δ_ ceramics for (**a**) *x* = 0.05, (**b**) 0.10, (**c**) 0.15, and (**d**) 0.20 calcined at (i) 1100 *°C* for 4 *h* and sintered at (ii) 1600 *°C* for 8 *h*.

The structural phase investigation of calcined powders and sintered pellets of Ho-substituted BaZrO_3_ were carried out using XRD technique, and their XRD patterns are presented in Figure 1(a–d). The XRD patterns of all Ho-substituted samples calcined at 1100 °C exhibit no impurity phase. The consequences of XRD are consistent with the corresponding DTA-TGA results, where negligible mass loss is observed above 1100 °C (Figure S1). Furthermore, assuming a cubic crystal system with $Pm\bar{3}m$ space group in all the Ho-substituted samples calcined at 1100 °C and sintered at 1600 °C, we have completed the Rietveld refinements of these XRD patterns. Hence, the refined patterns are made as a solid line in the respective Figure 1(a–d) ^37^. The distinguished structural parameters are tabulated in Table S1.

Analyzing all XRD patterns, it is manifested that flash pyrolysis process is very effective to prepare the pure phase of Ho-substituted BaZrO_3_ ceramics. The lattice parameter (Table S1), unit cell volume, Zr/Ho-O and Ba-O bond lengths are increased with substitution of Ho^3+^ in BaZrO_3_. This is in accordance with high ionic radius of Ho^3+^ (~90.1 *pm*) compare to Zr^4+^ (~72 *pm*). Furthermore, the crystallinity of all samples is decreased with the increase of Ho substitution in BaZrO_3_ samples (Table S1) may be due to lower diffusion rates of the cation with an increase of Ho-substitution in BaZrO_3_. The unit cell parameters extract from XRD patterns through Rietveld refinement method corresponding to *x* = 0.05, 0.10, 0.15 and 0.20 for BaZr_1-*x*_Ho_*x*_O_3-δ_ samples afterward sintered at 1600 °*C* were 4.1972, 4.1992, 4.2034 and 4.2057 *A°*, respectively. Therefore, it can be conjectured that the amount of Ho was completely incorporated at Zr-site of BaZrO_3_ perovskite, and its effective negative charge is completely counterbalanced by oxygen vacancies. The corresponding shift in the XRD peaks in the direction of the lower diffraction angles with the increase of Ho-substitution representing an increment of volume of the unit cell is apparent from Figure S2, also.

**Figure 2:**
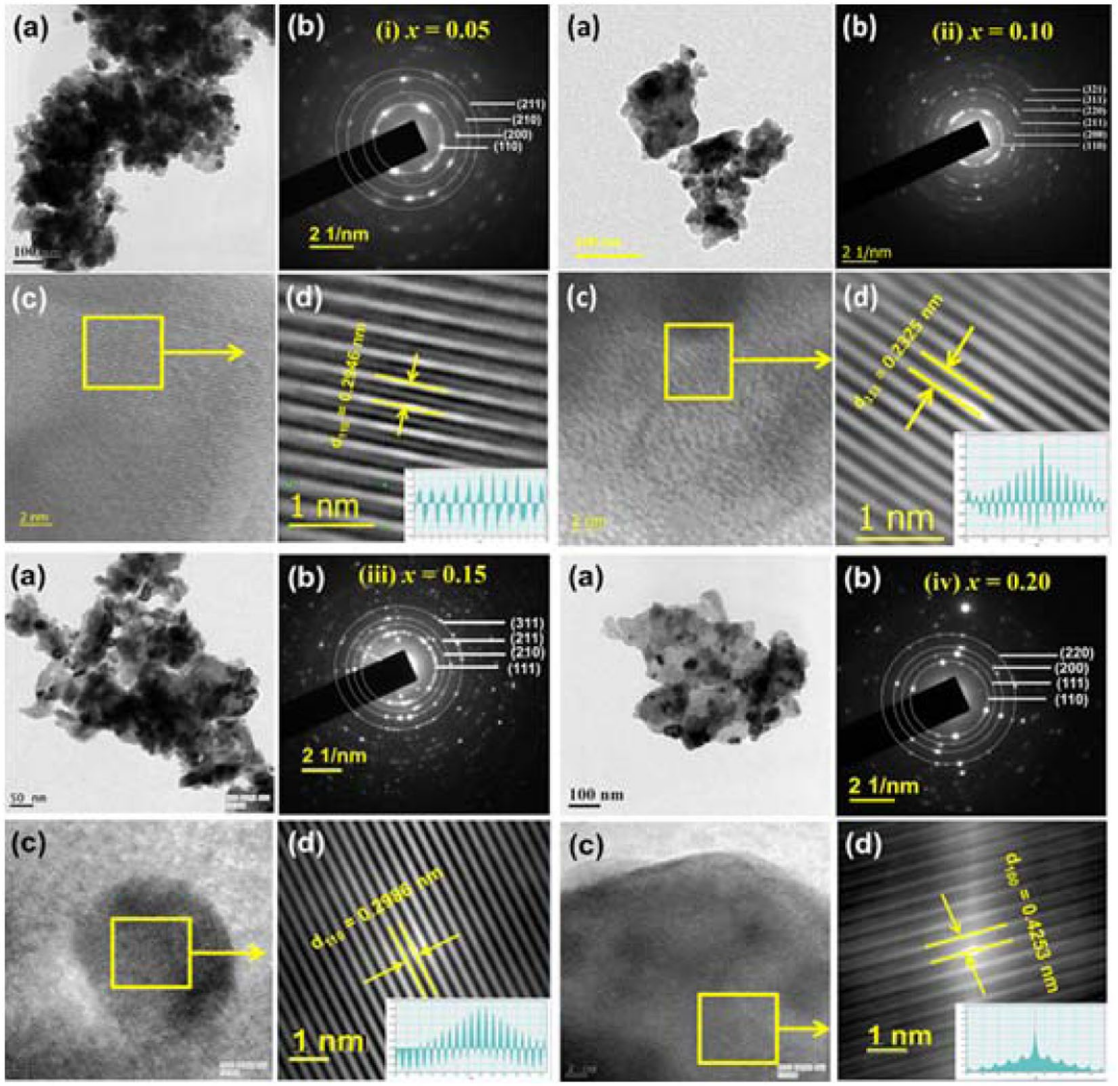
(**a**) A typical bright field TEM image, (**b**) corresponding selected area electron diffraction pattern (SAED), (**c**) lattice plane and (**d**) the simulated lattice plane of BaZr_1-*x*_Ho_*x*_O_3-δ_ ceramic nano-particles for (i) *x* = 0.05, (ii) 0.10, (iii) 0.15, and (iv) 0.20 calcined at 1100 °*C* for 4 *h*.

The microstructural investigation was carried out using HRTEM study. The Figuge 2(i–iv) displays (a) a typical bright field HRTEM image, (b) corresponding selected area electron diffraction (SAED) pattern, (c) lattice plane, and (d) simulated lattice plane for BaZr_1-*x*_Ho_*x*_O_3-δ_ (*x* = 0.05, 0.10, 0.15, and 0.20) nano-particles calcined at 1100 °*C* for 4 *h*, respectively. The HRTEM images of BaZr_1-*x*_Ho_*x*_O_3-δ_ (*x* = 0.05, 0.10, 0.15, and 0.20) samples reveal that particles are in sub-micrometer aggregation of nanocrystallites. The shape and size of particles are changed as substitution of Ho in BaZrO_3_ ceramics increases. The proof of crystal symmetry of BaZr_1-*x*_Ho_*x*_O_3-δ_ (*x* = 0.05, 0.10, 0.15, and 0.20) nano-particles was provided by using the SAED pattern as shown in Figure 2{i(b)–iv(b)}. In this figure, rings with spots are a well-known sign of the polycrystalline nature of all Ho-substituted BaZrO_3_ samples. It can be indexed with the plane of pure cubic crystal system for $Pm\bar{3}m$ space group. The orientation of the lattice plane remains established using simulated lattice pattern of square lattice plane image, as shown in Figure 2{i(d)–iv(d)}. The difference between two consecutive peaks confirms the lattice plane spacing (*d*_*hkl*_). The lattice plane spacing (*d*_*hkl*_) of all samples calculated from the simulated lattice pattern is comparable with lattice plane spacing (*d*_*hkl*_) estimated from the XRD pattern of all respective samples, as shown in Table 1.

**Table 1:** A comparison of the lattice planar spacing (*d*_*hkl*_) obtained from XRD and SAED pattern of BaZr_1-*x*_Ho_*x*_O_3-δ_ ceramics for *x* = 0.05, 0.10, 0.15, and 0.20 calcined at 1100 *°C* for 4 *h* in air.

*x*	Lattice constant obtained from XRD Pattern (*nm*)	Lattice plane (*hkl*)	Lattice planar spacing (*d*_*hkl*_) obtained from XRD pattern (*nm*)	Lattice planar spacing (*d*_*hkl*_) obtained from SAED pattern (*nm*)
0.05	4.1981	110	0.2958	0.2946
0.10	4.2021	111	0.2326	0.2345
0.15	4.2052	110	0.2971	0.2988
0.20	4.2068	100	0.4233	0.4253

**Figure 3:**
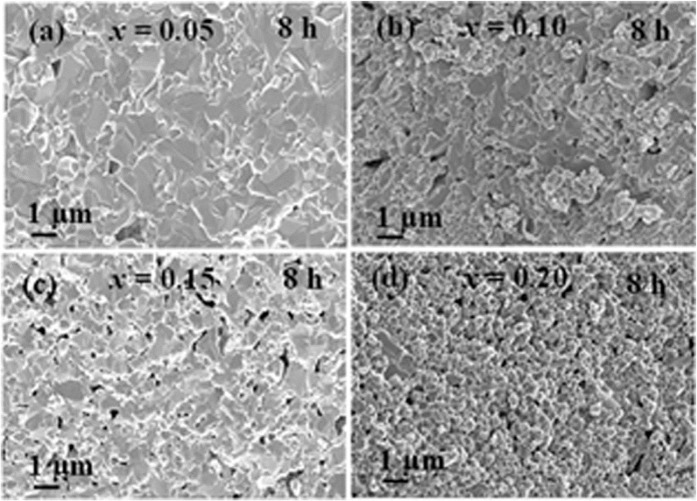
FESEM micrographs from fracture surface of BaZr_1-*x*_Ho_*x*_O_3-δ_ ceramics for (**a**) *x* = 0.05, (**b**) 0.10, (**c**) 0.15, and (**d**) 0.20 sintered at 1600 *°C* for 8* h* in air, respectively.

Figure 3(a–d) exhibits the FESEM images of fracture surface for a pellet of BaZr_1-*x*_Ho_*x*_O_3-δ_ (*x* = 0.05, 0.10, 0.15, and 0.20) samples sintered at 1600 °*C* for 8 *h* in air. The FESEM images exhibit that bimodal type of grains in all the samples except for *x* = 0.20. Furthermore, the average grain size is decreased with the increase of Ho-substitution in BaZrO_3,_ as displayed in Table 2 (Figure S3). Therefore, a decrease in average grain size recommends the lower diffusion rates of the cation with an increment of Ho-substitution in BaZrO_3_^38,39^.

**Table 2:** Average grain size from facture surface and relative density of BaZr_1-*x*_Ho_*x*_O_3-δ_ ceramics for *x* = 0.05, 0.10, 0.15, and 0.20 sintered at 1600 *°C* for 8 *h* in air.

*x*	Average grain size (*μm*)		Relative density (%)
	Small grain	Large grain	
0.05	0.43	1.18	94.7
0.10	0.34	1.04	93.5
0.15	0.26	0.82	92.6
0.20	—	0.73	91.3

All the sintered pellets at 1600 °*C* for 8 *h* have relative densities of >91%, as shown in Table 2. The Ho-substituted BaZrO_3_ sintered samples at 1600 °*C* for 8 *h* have microstructural mixtures of coarse and fine grains, as shown in Figure S3 (Table 2) through the grain size distribution curve. The microstructures of Ho-substituted BaZrO_3_ samples are same as that of Y-substituted BaZrO_3_ due to comparable ionic radius (Ho = 90.1 *pm* and Y = 90 *pm*), whose microstructure is the combination of fine and coarse grains as reported earlier^34^. This type of microstructure is due to the mixture of the different phases at a particular condition of synthesis and sintering temperature. The mixture of different phases reveals that cations should diffuse over a large distance to achieve equilibrium phase at 1600 °*C*, while the sintering time for 8* h* is not sufficient for sintering to achieve equilibrium phase at 1600 °*C*^38^ .However, there is no evidence in appearance of two phases in XRD patterns of Ho-substituted BaZrO_3_ ceramics calcined at 1100 °*C* for 4 *h*.

### Raman studies

**Figure 4:**
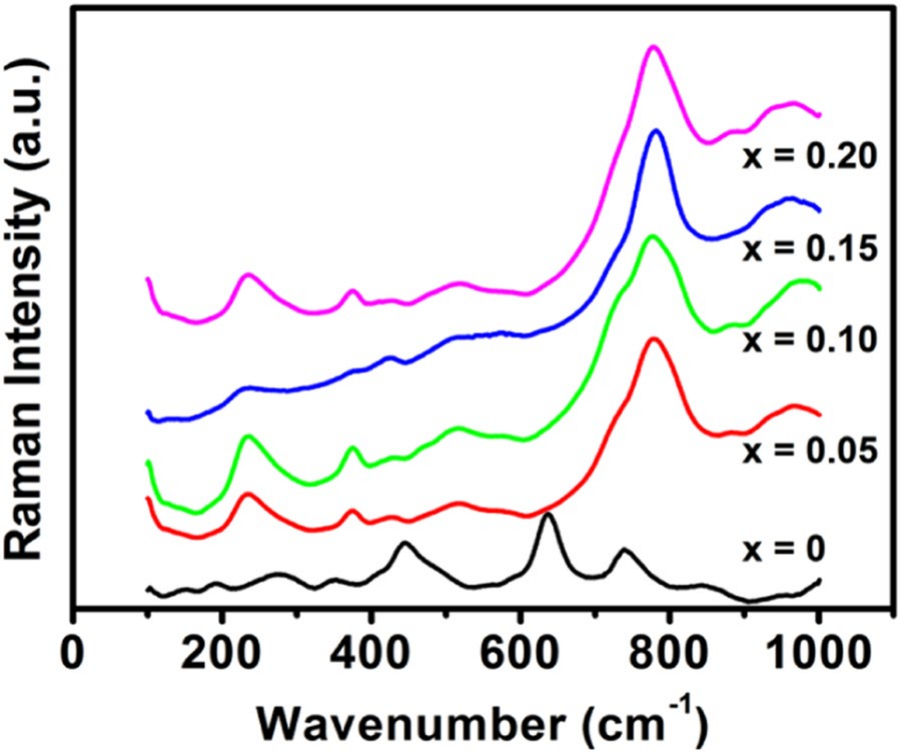
Raman spectra of BaZr_1-*x*_Ho_*x*_O_3-δ_ ceramics for *x* = 0, 0.05, 0.10, 0.15, and 0.20 sintered at 1600 *°C* for 8* h* in air, respectively.

Figure 4 exhibits Raman scattering spectra of BaZr_1-*x*_Ho_*x*_O_3-δ_ (*x* = 0, 0.05, 0.10, 0.15, and 0.20) ceramics sintered at 1600 °*C* for 8 *h* in air from 100 to 1000 *cm*^*−1*^ range. Generally, it is expected that Raman spectrum of ideal perovskite structure is featureless. But, there are several vibrational modes observed in all the samples. As there could be neither oxygen vacancies nor substituent atoms in BaZrO_3_, so it is expected that all observed active vibrational modes are only due to second-order scattering^40,41^. However, Karlsson *et al*.^42^ reported that vibrational mode around 200 *cm*^*−1*^ is associated with the torsional motion of lattice, which originates due to lattice distortion. Furthermore, Slodczyk *et al*.^42^ suggested that vibrational modes in Raman spectrum of BaZrO_3_ are mainly attributed to nanodomains having local symmetry different from that of cubic symmetry. As this spectrum consists of a broad band, so it is expected that distortions in cubic lattice to be small. In all samples, most of the bands exhibit some shifting and different intensity with the variation of composition. The appearance up of translation oscillation modes from 50 to 250 *cm*^*−1*^ is promoted by the motion of Ba^2+^ in the BaO_12_ cuboctahedra networks, which is dominated by Coulombic interactions^42–44^. At high frequencies, the peaks are mainly attributed to the mode of strongly covalent bonded oxygen in ZrO_6_ octahedral networks^40–42^. Therefore, the peaks from 300 to 500 *cm*^*−1*^ and 600 to 900 *cm*^*−1*^ range can be due to bending (*d*) and symmetric stretching (*n*) modes of oxygen bonds, more specially^43,44^.

In case of Ho-substituted BaZrO_3_ ceramics (*x* = 0.05, 0.10, 0.15 and 0.20), the vibrational band from 250 to 900 *cm*^*−1*^ range is associated to distortion of Zr/HoO_6_ octahedra because of tilting of Zr/HoO_6_ octahedra and a slight distortion of Zr/HoO_6_ in the direction of c-axis. This distortion in octahedra is mainly due to accommodation of larger size of Ho^3+^ atom (90.1 *pm*) at Zr-site (72 *pm*) and formation of oxygen vacancies in BaZrO_3_^41–44^. The weak vibration band around 130 *cm*^*−1*^ is assigned to deformational motion and stretching vibrations of Ba-Zr/HoO_6_^41^. This band is the signature of structure of Ho-substituted BaZrO_3_ perovskite and indicates a cubic structure of perovskite without any structural phase transition^42^. The vibration bands from 200 to 900 *cm*^*−1*^ range become more intense and broad as substituent concentration in BaZrO_3_ increases. It reveals that local deformations become more pronounced without changing the inclusive feature of spectra as Ho-concentration increases. The long-range average cubic structure remains similar as observed in XRD patterns. The distortion in the perovskite structure of pure and Ho-substituted BaZrO_3_ ceramics are completely diverse from each other. It can be identified by the shift of mode near 250 to 350 *cm*^*−1*^ towards the lower wave number for *x* = 0 to *x* = 0.20 substituent content and established to change in the tilt angle of ZrO_6_ octahedra^45^.

### Electrical impedance analysis

**Figure 5:**
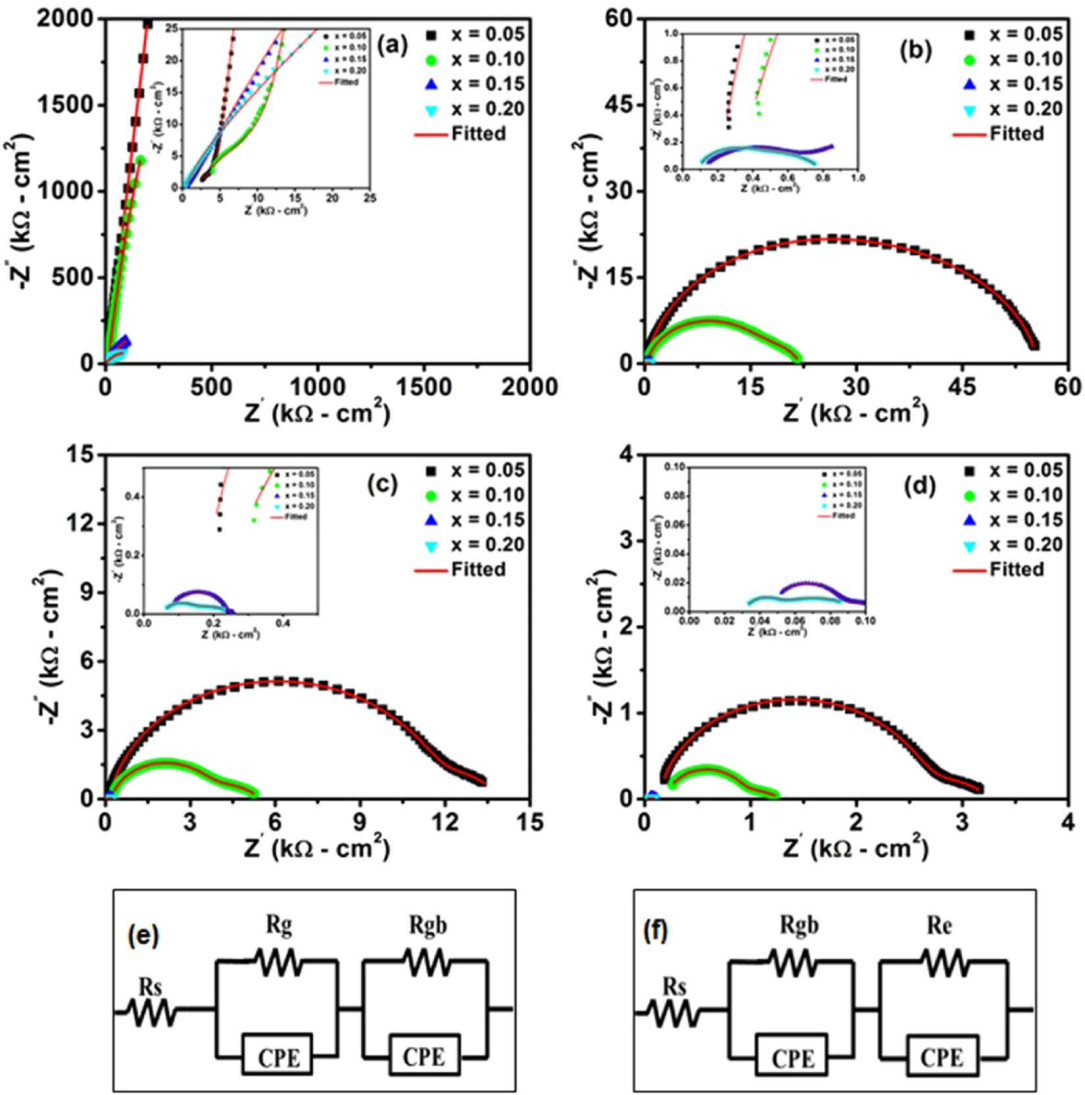
Complex impedance spectra (Nyquist plots) of BaZr_1-*x*_Ho_*x*_O_3-δ_ ceramics for *x* =  0.05, 0.10, 0.15 and 0.20 at (**a**) 300 *°C*, (**b**) 600 *°C*, (**c**) 700 *°C*, and (**d**) 800 *°C* in 3% humidified O_2_ atmosphere, respectively. Electrical equivalent circuits used to fit impedance data of Ho-substituted BaZrO_3_ samples, (**e**) circuit fits the impedance data at 300 *°C* and (**f**) circuit fits the impedance data at 600, 700, and 800 *°C*, respectively.

The impedance spectra (Nyquist plots) of BaZr_(1-*x*)_Ho_*x*_O_3-δ_ (*x* = 0.05, 0.10, 0.15, and 0.20) ceramics in 3% humidified O_2_ atmosphere at 300, 600, 700, and 800 °*C*, respectively are offered in Figure 5(a–d), where equivalent electric circuit are employed for the analysis of impedance data. For calculation of resistance, the ZSimpWin 3.21 software is exploited for impedance spectra fitting and corresponding equivalent electric circuit is displayed in Figure 5(e,f). Out of the two circuits shown, the circuit displayed in Figure 5(e) fits the data at 300 °*C* temperature, meanwhile the circuit in Figure 5(f) fits the data at 600, 700, and 800 °*C* temperature, respectively. In these electric circuits, *R*_*g*_, *R*_*gb*_ and *R*_*e*_ signify the grain (bulk), grain-boundary and electrode resistance, respectively. Furthermore, the *R*_*s*_ and *CPE* characterize series resistance and constant phase element, respectively.

The complex impedance spectra from Nyquist plots for all samples at 300 °*C* in 3% humidified O_2_ atmosphere exhibits the existence of two depressed semicircular arcs. It reveals that two types of relaxation phenomena with sufficiently different relaxation times occurred in the materials at 300 °*C*. The low resistive arc at high frequency regime and high resistive arc at low frequency region are attributed to contribution from grain (bulk) interior and grain boundary, respectively. Furthermore, complex impedance spectra for all samples at 600, 700 and 800 °*C* exhibit again two depressed semicircles. However, the meanings of semicircular arcs are unlike from complex impedance spectra at 300 °*C*. The semicircle at high frequency side is possibly due to contribution from grain boundary and semicircle at low frequency side may be attributed to electrode polarization^46^. Furthermore, the capacitance value associated with bulk interior, grain boundary, and electrode polarization is found to around 10^−11^, 10^−9^, and 10^−6^ *F*, respectively^27^. The shape of semicircular arcs differs with respect to Ho-content and temperature, while the radii of these semicircular arcs decrease as such Ho-content and temperature increase. This observation exhibits the variation in resistive and capacitive part of the material. At high temperature, the contribution from grain in complex impedance spectra for all samples disappears. This may be attributed to high values of bulk relaxation frequency, preventing the appearance of arc at high temperature^47,48^. Thus, grain boundary effect dominates in all samples at high temperatures^49–51^.

### The total electrical conductivity of Ho-substituted BaZrO_3_ ceramics

It is considered from the past few years that there would be promising solid oxide fuel cell applications if advanced intermediate temperature proton conductors would be built up. In this context, the total electrical conductivity of Ho-substituted BaZrO_3_ has been attempted in 3% humidified atmosphere. The Ho-substitution on Zr-site in BaZrO_3_ promotes to form an oxide ion vacancy as provided in Eq. (3) through Kröger-Vink notation.

3 $$2Z{r}_{Zr}^{x}+{O}_{O}^{x}+H{o}_{2}{O}_{3}\to 2H{o{\prime} }_{Zr}+{V}_{O}^{\bullet \bullet }+2Zr{O}_{2}$$

**Figure 6:**
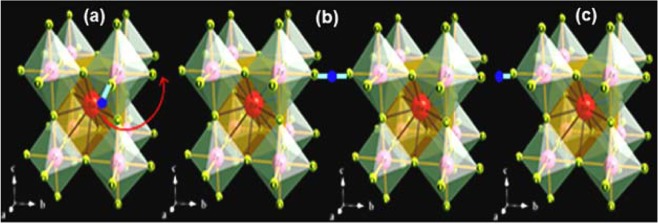
Schematic representation of the Grotthus mechanism for proton conduction.

The water molecule from vapor phase split up into $O{H}^{-}$ and ${H}^{+}$ ions. The $O{H}^{-}$ ion fills up an oxide ion vacancy, and the ${H}^{+}$ helps to form an $O{H}^{-}$ ion with oxygen lattice as specified in Eq. (2). As a result, the protons are induced by filling oxygen vacancies created by the substitution of the trivalent cation (Ho) into Zr site in BaZrO_3_ ceramics from 300 to 600 °C ranges. Thus, the conducting species may be proton (*H*^+^), oxide ion vacancy and hole in wet conditions^52^, which depends upon the gas atmosphere, and temperature, etc. Furthermore, the conduction of proton in such materials takes place according to the Grotthus mechanism. In this mechanism, proton conduct through a diffusion process, which is the combination of molecular reorientation around oxygen and the hopping of proton from oxygen to nearest neighbour oxygen (Figure 6)^53,54^.

**Figure 7:**
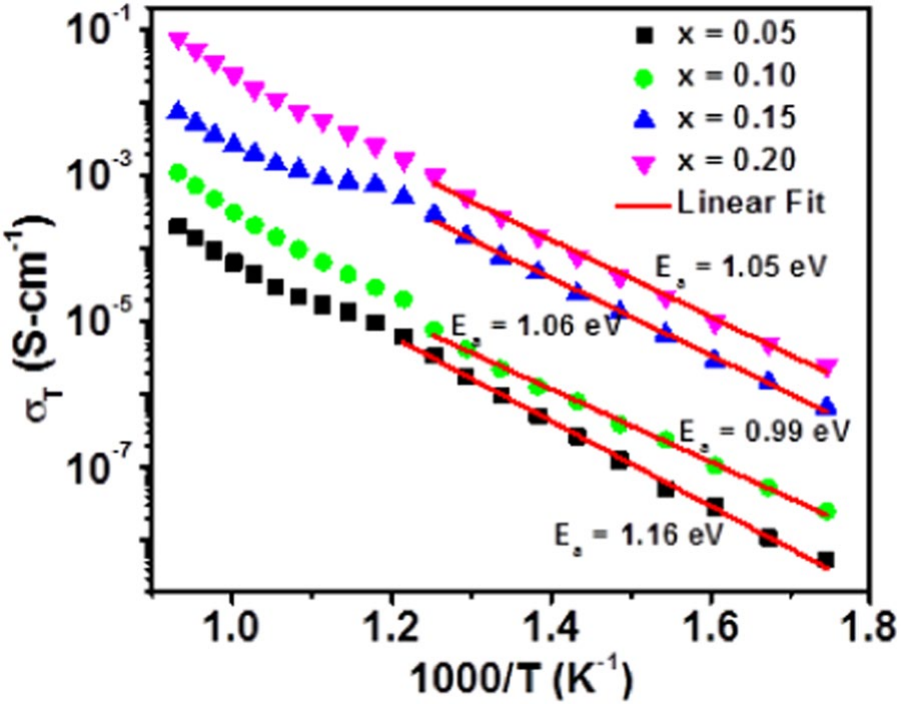
The total electrical conductivity of BaZr_1-*x*_Ho_*x*_O_3-δ_ ceramics for *x* = 0.05, 0.10, 0.15, and 0.20 in 3% humidified O_2_ atmospheres.

The total electrical conductivities were deduced in terms of resistance determined from the impedance data through accounting for sample geometry according to Eq. (4).

4 $$\sigma =\frac{L}{A}(\frac{1}{Rg+Rgb})$$

where, *L* is the sample thickness, *A* is area, *R*_*g*_ and *R*_*gb*_ are grain (bulk) and grain boundary resistance, respectively. At higher temperature, the semi-circular arc due to grain (bulk) interior and grain boundary cannot be clearly resolved. Therefore, the total resistance of the material can be calculated from intermediate frequency intercept of complex impedance spectra (corresponding to contribution from grain boundary) with the real axis at high temperature region. Furthermore, the total electrical conductivity of BaZr_1-*x*_Ho_*x*_O_3-δ_ (*x* = 0.05, 0.10, 0.15, and 0.20) samples in 3% humidified O_2_ atmospheres is shown in Figure 7. The BaZr_1-*x*_Ho_*x*_O_3-δ_ sample for *x* = 0.20 exhibits highest total electrical conductivity in 3% humidified atmosphere as revealed in Figure 7. Table 3 presents a detailed comparison between conductivities obtained under the present study and previous reports.

**Table 3:** Comparison of the total electrical conductivity for BaZr_1-*x*_M_*x*_O_3-δ_ (M = Sc, In, Lu, Yb, Tm, Er, Y, Ho, Gd, Sm and Nd) proton-conducting electrolyte ceramics.

M	Ionic radius (*pm*)	*x*	Conductivity (*S-cm*^*−1*^)	Activation energy (*eV*)	Sintering conditions	Measurement conditions	Ref.
Sc	74.5	0.20	2.90 × 10^−4^ at 500 °*C*	0.98	1600 °*C*/8 *h* in air	Humidified atmosphere	^26^
In	80.0	0.20	6.90 × 10^−4^ at 600 °*C*	0.89	1600 °*C*/8 *h* in air	Humidified atmosphere	^26^
		0.75	1.00 × 10^−4^ at 600 °*C*	0.81	1325 °*C*/48 *h* in air	Dry Ar atmosphere	^33^
Lu	86.0	0.20	2.80 × 10^−5^ at 400 °*C*	0.90	1600 °*C*/8 *h* in air	Humidified atmosphere	^26^
Yb	86.8	0.20	1.10 × 10^−2^ at 600 °*C*	—	1600 °*C*/24 *h* in O_2_	5% H_2_O – H_2_ atmosphere	^58^
		0.10	5.60 × 10^−5^ at 550 °*C*	—	1500 °*C*/48 *h* in air	Dry air	^32^
Tm	88.0	0.20	1.13 × 10^−2^ at 600 °*C*	—	1600 °*C*/24 *h* in O_2_	5% H_2_O – H_2_ atmosphere	^58^
Er	89.0	0.20	1.36 × 10^−2^ at 600 °*C*	—	1600 °*C*/24 *h* in O_2_	5% H_2_O – H_2_ atmosphere	^58^
Y	90.0	0.20	5.80 × 10^−4^ at 550 °*C*	0.86	1600 °*C*/8 *h* in air	Humidified atmosphere	^26^
		0.20	2.29× 10^−2^ at 600 °*C*	—	1600 °*C*/24 h in air	H_2_O saturated N_2_ atmosphere	^39^
		0.20	1.39 × 10^−2^ at 600 °*C*	—	1600 °*C*/24 *h* in O_2_	5% H_2_O – H_2_ atmosphere	^58^
Ho	90.1	0.05	2.96 × 10^−5^ at 650 °*C*	1.16	1600 °*C*/8 *h* in air	3% humidified O_2_ atmosphere	Present work
		0.10	1.42 × 10^−4^ at 650 °*C*	0.99			
		0.15	1.43 × 10^−3^ at 650 °*C*	1.06			
		0.20	1.11 × 10^−2^ at 650 °*C*	1.05			
Gd	93.8	0.20	1.80 × 10^−4^ at 600 °*C*	0.74	1600 °*C*/8 *h* in air	Humidified atmosphere	^26^
Sm	95.8	0.20	8.90 × 10^−5^ at 600 °*C*	0.96	1600 °*C*/8 *h* in air	Humidified atmosphere	^26^
Nd	98.3	0.07	2.01 × 10^−5^ at 628 °*C*	—	1700 °*C*/1 *h* in vacuum	Humid air	^59^

In general, the wet atmosphere boosts up the total electrical conductivity of the material significantly at the temperature region of 300 to 600 °C and oxygen partial pressure^55,56^. The overall electrical conductivity of the material also depends upon several factors such as charge carrier concentration, nature of moving charge carrier, mobility of charge carrier, etc. In 3% humidified O_2_ atmosphere, the hydration of BaZr_(1-*x*)_Ho_*x*_O_3-δ_ (*x* = 0.05, 0.10, 0.15, and 0.20) lattice by H_2_O molecules lead to remarkable proton conductivity, as shown through Eq. (2). At low temperatures (<600 *°C*), the reaction equilibrium presented by Eq. (2), is most favourable for proton formation. This signposts that the total conductivity may be dominated by conduction of proton in the samples. The total electrical conductivity in 3% humidified O_2_ atmosphere is increased with the addition of amount of Ho-content throughout the temperature ranges (Figure 7). An increase in the total electrical conductivity may be due to proton, which is the majority charge carrier in 3% humidified O_2_ atmosphere and increased with the increase of Ho-concentration. Furthermore, ionic radius of Ho^3+^ (90.1 *pm*) is larger than that of Zr^4+^ (72 *pm*), which expands the unit cell of BaZrO_3_ and volume of the unit cell with the increasing amount of Ho-content in BaZrO_3_ ceramics. This leads to enhance the width of migration channel for proton conduction. The grain boundary conductivity is dominating (Nyquist plots) in a wet oxidizing atmosphere at all elevated temperature because of core-space charge layer in the grain boundary, which give rise to the depletion of positive charge in the layer near to boundary core^57^. Furthermore, the activation energy for all samples is higher than 1 eV under 3% humidified O_2_ atmosphere. However, the activation energy is higher than typically observed for proton conduction in Y-substituted BaZrO_3_ ceramics (typically 0.45–0.55 *eV*). This discrepancy may be attributed to partial hydration of samples in 3% humidified O_2_ atmosphere and the total electrical conductivity is probably mainly due to the conduction of both proton and oxygen vacancy. The total electrical conductivity obtained in such an environment is 5.8 × 10^−3^ S-cm^−1^ at 600 °*C* for BaZr_0.8_Ho_0.2_O_3-δ_. This is almost the same order to that of Y, Yb, Tm, Er, Ho-substituted BaZrO_3_ ceramics sintered at 1600 °*C* for 24 *h* as accessible in Table 3. Furthermore, the total conductivity of BaZr_0.8_Ho_0.2_O_3-δ_ sample is one or two orders greater than that of Sc, In, Lu, Y, Gd, and Sm-substituted BaZrO_3_ ceramics sintered at 1600 °*C* for 8 *h*^26,32,33,38,58,59^. The variation of the total electrical conductivity with the concentration of Ho-substitution is revealed in Figure S4. The total electrical conductivity measured at 600 *°C* for *x* = 0.20 is nearly three orders higher than that for *x* = 0.05 concentration. At higher temperature (>600 °*C*), the slope of Arrhenius plot changes in the conductivity plots. This indicates that the conductivity is attributable to conduction of hole or oxide ion, and not for the conduction of proton. Bohn and Schober have also stated similar findings for Y-doped BaZrO_3_ perovskites^9^. However, an increase in the total conductivity at higher temperatures, under O_2_ environment settles that hole conduction is dominating, according to the following reaction:

5 $$1/2{O}_{2}(g)+{V}_{O}^{\bullet \bullet }\leftrightarrow {O}_{O}^{x}+2{h}^{\bullet }$$

## Conclusions

The highly pure and Ho-substituted BaZrO_3_ nano-sized powders were synthesized through a flash pyrolysis process and followed by conventional sintering. The Rietveld refined XRD pattern approves that all Ho-substituted BaZrO_3_ samples sintered at 1600 *°C* revealing $Pm\bar{3}m$ space group of cubic crystal system. The densification and average grain size of all samples are initiated to decrease with the increase of Ho-substitution in BaZrO_3_ ceramics. This is mainly because of the lower diffusion rate of cation as Ho-substitution in BaZrO_3_ ceramics increases. Furthermore, the samples exhibit non-uniform electrical microstructure in all probing range of temperature and frequency. The electrical microstructure from grain (bulk) interior and grain boundary is detected from 100 *Hz* - 1 *MHz* frequency range for different temperatures. Complex impedance spectra from Nyquist plots reveal that the effect of grain boundary dominates in all over the samples, which is due to the formation of the potential barrier. The total conductivity is increased with the intensification of substitution of Ho-content in BaZrO_3_ ceramics. This is because of the creation of additional oxygen vacancies. The total estimated electrical conductivity of BaZr_0.8_Ho_0.2_O_3-δ_ sample has arrived to be 5.8 × 10^−3^ S-cm^−1^ at 600 °*C*. Therefore, flash pyrolysis route is a most robust technique to synthesize highly pure and nano-sized BaZr_1-*x*_Ho_*x*_O_3-δ_ (*x* = 0.05, 0.10, 0.15, and 0.20) ceramics powder, which may be assisted to improve the electrical conductivity of electrolyte ceramics for SOFCs.

## Methods

### Chemicals

Unless otherwise stated, nano-sized BaZr_1-*x*_Ho_*x*_O_3-δ_ (*x* = 0.05, 0.10, 0.15 and 0.20) powders were prepared by flash pyrolysis method using high purity (>99%) raw materials: Ba(NO_3_)_2_, ZrOCl_2_.8H_2_O, Ho(NO_3_)_3_.5H_2_O (Alfa Aesar).

### Powder preparation

At first, ZrOCl_2_.8H_2_O was added in deionized water, and then, it was precipitated as hydrated hydroxides after addition of liquor NH_4_OH. The precipitate was washed repeatedly with deionized water to remove chloride ions. After that, the resultant precipitate was dissolved in dilute HNO_3_ solution. A clear aqueous solution of Ba(NO_3_)_2_ and Ho(NO_3_)_3_.5H_2_O was prepared by adding distilled water in required proportion. The stoichiometric amount of aqueous solutions was mixed in 2000 *mL* beaker using a magnetic stirrer, and the desired amount of citric acid was added to metal ions in the above solution. The ratio of citric acid and metal ions was maintained as 1.5:1 proportion. The pH of the above solution was adjusted at 7 using dilute NH_4_OH solution. Furthermore, the calculated amount of glycine (metal ions: glycine = 1:0.5) and ethylene glycol (metal ions: ethylene glycol = 1:1.5) were also added to it. Initially, the citric acid and glycine are played a role of chelating agents, and finally, both are functioned as a fuel during the combustion. The resultant solution was placed in a pit furnace at 350 °*C* with concurrent IR heating from the top. The water was slowly evaporated, leading to form a gel. Finally, the gel was changed into a low-density black fluffy mass because of sudden combustion in the gel. To derive Ho-substituted BaZrO_3_ ceramics in the form of nano-sized particles, as-prepared powder containing carbon and other organic impurity obtained from flash pyrolysis route was calcined at 1100 °*C* in the alumina crucible for 4 *h* in air. During heating, the organic part present in as-prepared powder was burnt out in the air to form a nano-sized powders. The quality and formation of the phase of ceramic materials were checked by the XRD pattern.

### Preparation of bulk sample

**Figure 8:**
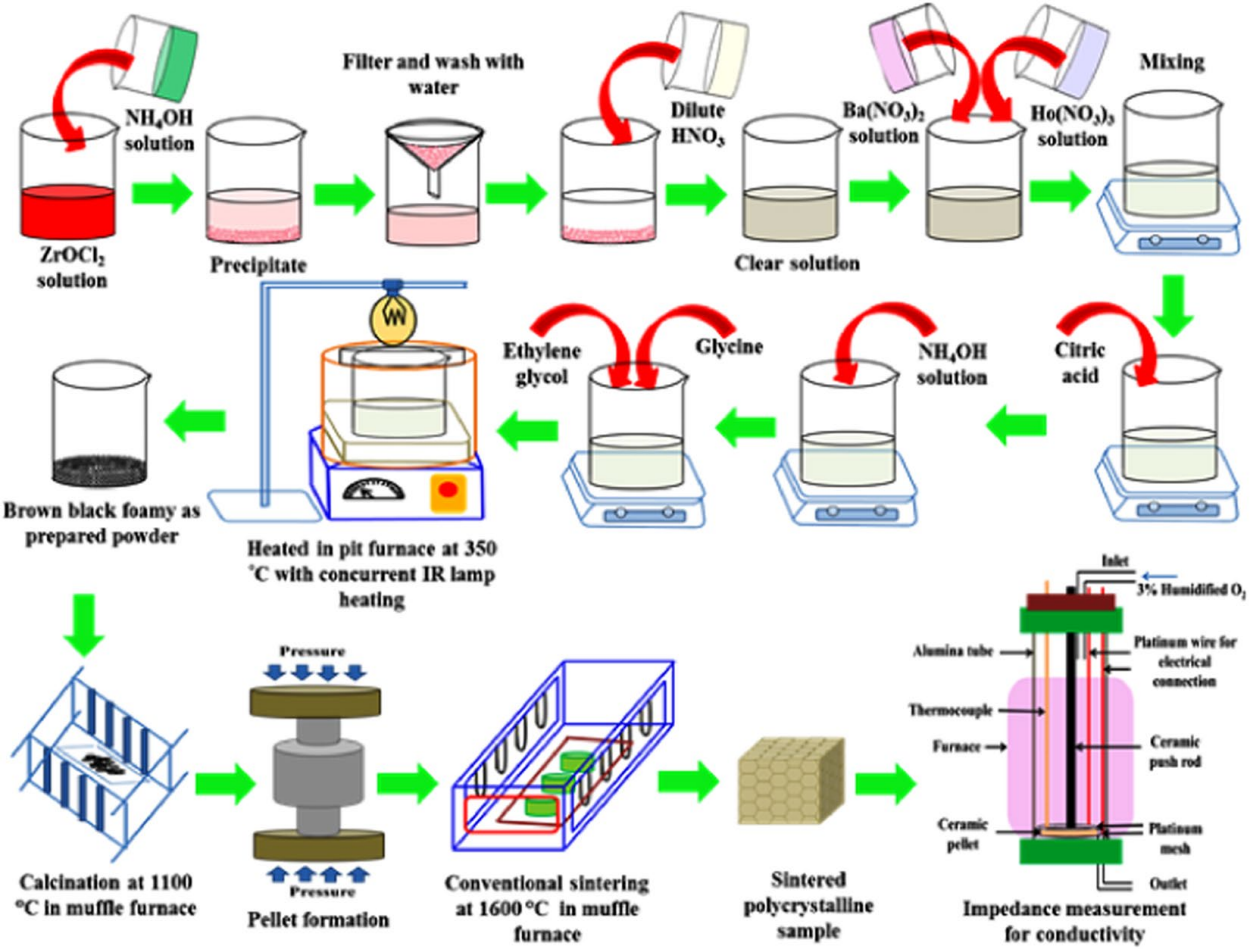
Schematic of processing of Ho-substituted BaZrO_3_ ceramic by a flash pyrolysis process followed by a conventional sintering, and the obtained conductivity through impedance measurement.

The BaZr_1-*x*_Ho_*x*_O_3-δ_ (BZH) powders calcined at 1100 °*C* for 4 *h* were crushed to obtain very fine powder using agate mortar and pestle for 15 *min*. The binder solution {(polyvinyl alcohol (PVA)} was mixed in crushed powder to obtain better compactness among granules of the materials and pressed uniaxially in a steel die under the pressure of 340 *MPa* for 1 *min* in a hydraulic press. After attaining the pellet form of Ho-substituted BaZrO_3_ perovskite powder using a hydraulic press, it is still required to densify compacted powder samples (green bodies) with continuous 3D structure. The green pellets of BaZr_1-*x*_Ho_*x*_O_3-δ_ were sintered at 1600 °*C*. But, as the evaporation of Ba occurs at such high temperature, the green pellets were hence covered with 10% extra of BaO during sintering to compensate evaporation of Ba from the bulk of pellet. All green pellets were sintered at 1600 °*C* for sintering time of 8 *h*. Schematic of processing for Ho-substituted BaZrO_3_ ceramics by a flash pyrolysis technique followed by a conventional sintering, and the obtained conductivity through an impedance measurement are shown in Figure 8. The sintering was carried out to get dense pellets for proton-conducting electrolyte in SOFCs application. The sintering profile is also displayed in Figure S5.

### Material characterization

The X-ray diffraction (XRD) (Bruker D8 Advance) was employed to investigate the information regarding phase and structure features of all samples. The XRD patterns of all samples were recorded within the range of 20° to 120° with a step size of 0.019° and a scan rate of 0.064°/min. Furthermore, High-resolution transmission electron microscopy (HRTEM) (JEOL-JEM-2100) was employed to study the particle size, particle morphology, lattice plane, etc. of the powder calcined at 1100 °*C* for 4 *h*. The microstructure features (i.e. grain size, grain morphology, etc.) were investigated through Field emission scanning electron microscopy (FESEM) of Zeiss (Merlin-Gemini II). The distributions of grain size were estimated through ImageJ software for all the samples. The Raman spectroscopic measurement of all sintered samples at 1600 °*C* was carried out using double pre-monochromator (1800 grooves*/mm* grating) of Raman spectrophotometer (T64000, Horiba Jobin Yvon Ltd. USA) with 514.5 *nm* radiation of Ar^+^ laser.

### Electrical impedance measurements

To investigate the total electrical conductivity, a complex plane AC impedance spectroscopic study through a Frequency Response Analyzer (ModuLab, Solartron, UK) from 100 *Hz* to 1 *MHz* range was employed. For above measurement, platinum paste (Metalo) was painted for the electrode on both the polished surface (by SiC polishing paper) of circular pellets (diameter: 8 *mm*, and thickness: 1–2 *mm*) and followed by heating at 1000 *°C* for 2 *h* in the air to prepare as Pt-BaZr_1-*x*_Ho_*x*_O_3-δ_-Pt symmetric cell configuration. The AC amplitude was set at 300 *mV* and measurements were carried out in between 300 *°C* and 800 *°C* under 3% humidified O_2_ atmosphere.

## Supplementary information

Revised Supporting information.
