# Supplementary material for: A Promising Proton Conducting Electrolyte BaZr1-xHoxO3-δ (0.05 ≤ x ≤ 0.20) Ceramics for Intermediate Temperature Solid Oxide Fuel Cells
Source: Sci Rep. 2020 Feb 26;10:3461. doi: 10.1038/s41598-020-60174-4 (PMC7044191; doi:10.1038/s41598-020-60174-4)
Supplement: Supplementary file 1 — Revised Supporting information. [file 41598_2020_60174_MOESM1_ESM.pdf]

Supporting Information

A Promising Proton Conducting Electrolyte $BaZr_{1-x}Ho_xO_{3-\delta}$ ($0.05 \leq x \leq 0.20$) Ceramics for Intermediate Temperature Solid Oxide Fuel Cells

Deepash S. Saini[1*], Avijit Ghosh[2], Shuvendu Tripathy[3], Aparabal Kumar[3], Sanjeev K. Sharma[3], Nawnit Kumar[4], Shubhankar Majumdar[5] and Debasis Bhattacharya[3]

[1]*Department of Physics, Deen Dayal Upadhaya Gorakhpur University, Gorakhpur - 273009, India*

[2]*Department of Physics, Central University of Jharkhand, Ranchi - 835205, India*

[3]*Materials Science Centre, Indian Institute of Technology, Kharagpur - 721302, India*

[4]*Department of Physics, Malti Dhari College, Naubatpur, Patliputra University Patna, Patna - 801109, India*

[5]*Department of Electronics and Communication Engineering, National Institute of Technology, Meghalaya - 793003, India*

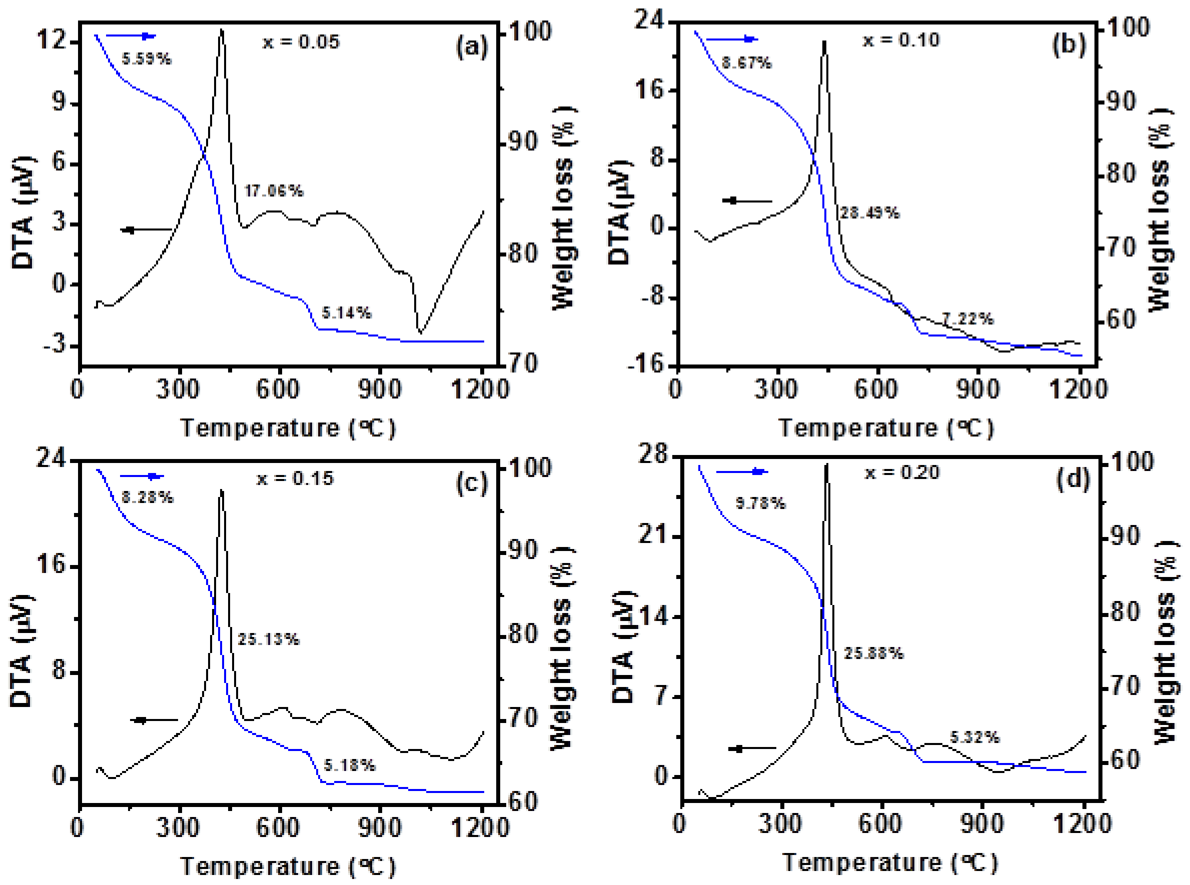

Figure S1: The thermal decomposition profile of the as-prepared $BaZr_{1-x}Ho_xO_{3-\delta}$ ceramics nano-sized powders for (a) x = 0.05, (b) 0.10, (c) 0.15, and (d) 0.20) in nitrogen atmosphere.

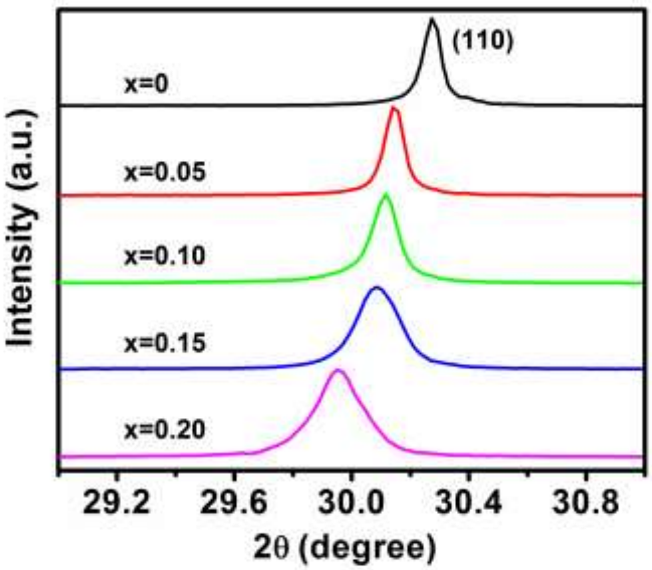

Figure S2: Shifted peak (110) position of $BaZr_{1-x}Ho_xO_{3-\delta}$ ($0.05 \le x \le 0.20$) samples sintered at 1600 ℃ for 8 *h* in air.

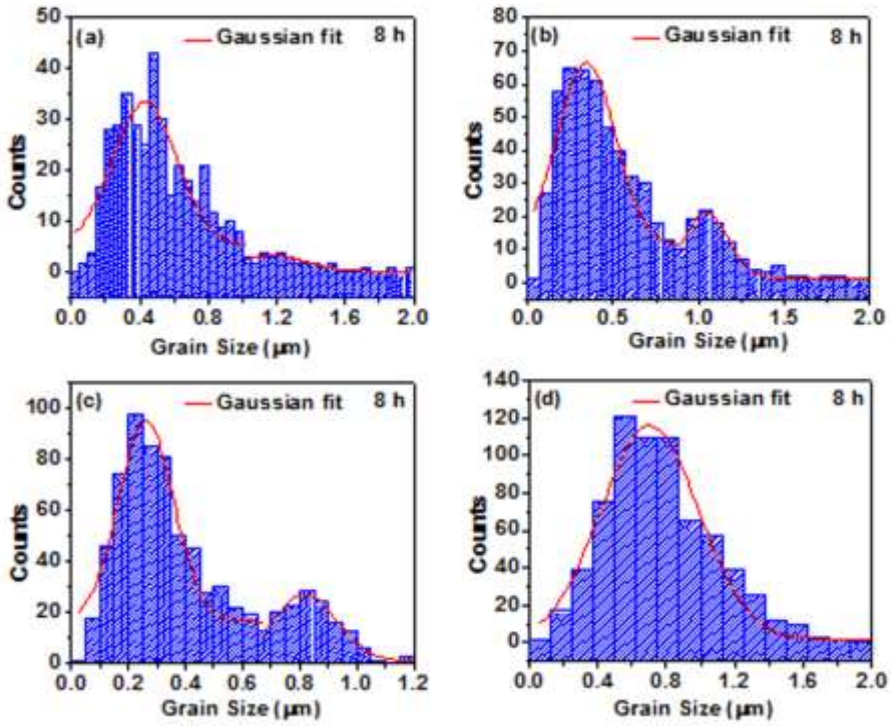

Figure S3: Grain size distribution of fracture surface of $BaZr_{1-x}Ho_xO_{3-\delta}$ ceramics for (a) x = 0.05, (b) 0.10, (c) 0.15, and (d) 0.20 sintered at 1600 °*C* for 8 *h* in air.

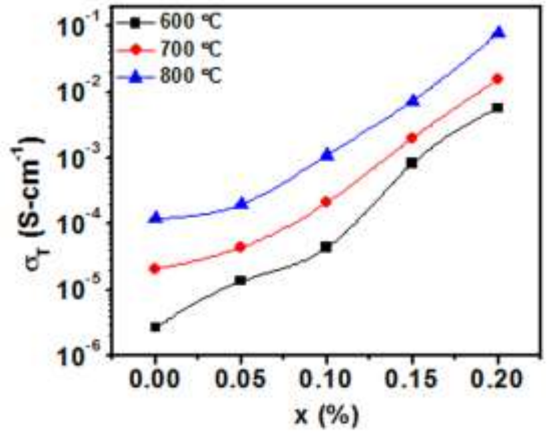

Figure S4: The variation of total conductivity with Ho-substitution in $BaZrO_3$ ceramics under 3% humidified O_2 atmosphere.

.

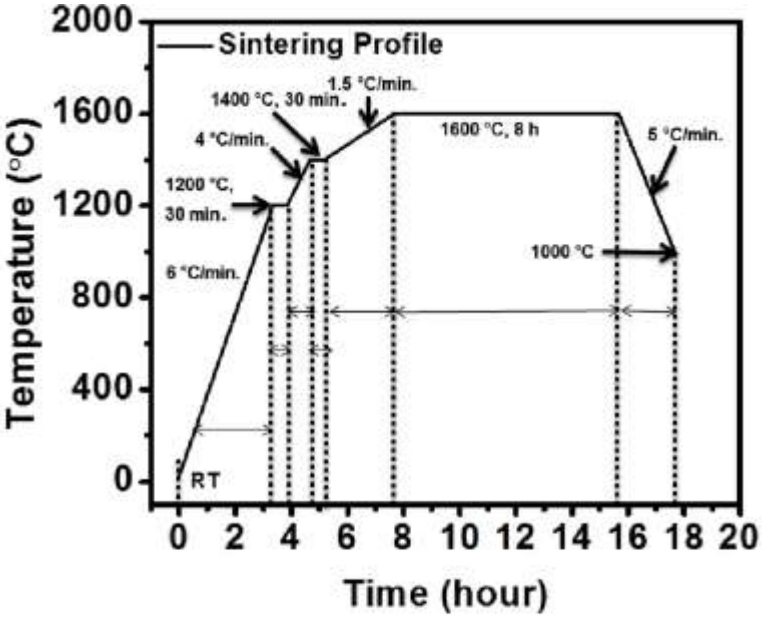

Figure S5: Sintering profile of Ho-substituted $BaZrO_3$ ceramics.

Table S1: Refined structural parameters of $BaZr_{1-x}Ho_xO_{3-\delta}$ ceramics nano-powders for x = 0.05, 0.10, 0.15, and 0.20 heated at several temperatures

x	T (ºC)	α (Å)	V_l ($Å^3$)	Refined Rietveld Parameters	Bond length (Å) [ZrO_6] Zr/Ho-O	[BaO_{12}] Ba-O	D (nm)
**0.05**	1100	4.1981	73.987	R_p = 9.96, R_{wp} =10.2, R_{exp} = 9.75, χ^2 = 1.09	2.0991	2.9685	34.27
	1600	4.1972	73.940	R_p = 11.2, R_{wp} =10.5, R_{exp} = 8.65, χ^2 = 1.45	2.0986	2.9678	42.84
**0.10**	1100	4.2021	74.199	R_p = 11.4, R_{wp} =9.45, R_{exp} = 7.74, χ^2 = 1.49	2.1011	2.9713	37.70
	1600	4.1992	74.046	R_p = 12.2, R_{wp} =11.9, R_{exp} = 9.71, χ^2 = 1.50	2.0996	2.9693	46.14
**0.15**	1100	4.2052	74.364	R_p = 14.2, R_{wp} =13.6, R_{exp} = 12.5,χ^2 = 1.19	2.1026	2.9735	37.68
	1600	4.2034	74.268	R_p = 11.5, R_{wp} =10.4, R_{exp} = 7.20, χ^2 = 2.10	2.1017	2.9723	47.11
**0.20**	1100	4.2068	74.448	R_p = 13.5, R_{wp} =12.9, R_{exp} = 10.6, χ^2 = 1.49	2.1034	2.9747	41.89
	1600	4.2057	74.390	R_p = 12.6, R_{wp} =11.5, R_{exp} = 7.47, χ^2 = 2.36	2.1029	2.9739	48.52

*R_p: Profile factor, R_{wp}: Weighted profile factor, R_{exp}: Expected weighted profile factor, χ^2: Goodness of fit.
.
